# Increasing demands on commissioning and quality assurance for cone-based multiple brain metastases robotic radiosurgery: evaluation in a custom phantom

**DOI:** 10.1007/s13246-026-01765-9

**Published:** 2026-07-21

**Authors:** Godfrey Mukwada, Pejman Rowshanfarzad, Martin A. Ebert

**Affiliations:** 1 Department of Radiation Oncology, Sir Charles Gairdner Hospital, Hospital Ave, Nedlands, WA 6009 Australia; 2 School of Physics, Mathematics and Computing, The University of Western Australia, Crawley, WA Australia; 3 Centre for Advanced Technologies in Cancer Research (CATCR), Perth, WA Australia

**Keywords:** Multiple brain metastases, Stereotactic radiosurgery, CyberKnife, Ray tracing, Monte Carlo, Commissioning

## Abstract

CyberKnife stereotactic radiosurgery (SRS) treatment of multiple brain metastases (MBM) using fixed cone collimation is well established. This study aims to rigorously evaluate the commissioning of MBM SRS using feasible complex clinical scenarios. Sixteen gross tumour volumes (GTVs) were contoured on the CT of an in-house developed phantom. Six treatment plans, testing different levels of complexity, were generated using the Ray Tracing (RT) dose calculation algorithm and recalculated with the Monte Carlo (MC) algorithm. Respective quality assurance (QA) plans were generated on the same phantom and delivered on a CyberKnife M6 system. QA was performed with EBT4 film inserted in the GTVs under investigation. The films were scanned and analysed using in-house developed software for comparison with RT and MC calculated dose distributions in the film planes. For simple cases, RT and MC performed equally well. As the number and proximity of GTVs increased, with complex GTV geometrical distribution and multiple prescription dose levels (MPDLs), the plan gamma pass rate at 3%/1 mm was 36.2% for RT compared to 96.4% for MC. Corresponding RT and MC calculated dose planes were also compared for the failing cases, and similar differences were observed. In conclusion, commissioning of the algorithm with simple and few brain metastases should not be assumed to hold for MBM SRS. The RT algorithm was found to be unsuitable for complex MBM SRS. When commissioning SRS techniques for treating multiple GTVs, the increased demands of proximity and geometrical distribution of GTVs, and the use of MPDLs, should be carefully considered.

## Introduction

Although comprehensive guidelines and protocols have been crafted for commissioning treatment planning systems (TPSs) [1–6], the tests should be dynamic and relevant to changes in treatment techniques and their complexity. The use of single or no isocentre techniques for multiple brain metastases (MBM) stereotactic radiosurgery (SRS) is increasing and, as such, relevant rigorous TPS commissioning tests are needed.

The CyberKnife (Accuracy Inc., Sunnyvale, CA, USA) comprises a robotic arm with six points of rotation, a mounted linear accelerator head, stereoscopic imaging and an infra-red tracking system. Radiation delivery can be through the multileaf collimator (MLC), iris (dodecagon-shaped field), or fixed cone beam collimation. The Precision TPS, commonly used for planning CyberKnife treatments, can use three algorithms for dose calculation, namely, Ray Tracing (RT), Monte Carlo (MC), and Finite Size Pencil Beam (FSPB). The RT algorithm uses relative electron density (RED) to determine the effective path length (EPL) for dose calculation and is limited in accounting for the dose from lateral scatter, which renders it generally less accurate in a heterogeneous medium. The MC is a probabilistic algorithm that traces each particle until it deposits the dose, and the interaction in each medium is well characterised. MC dose calculation accuracy is influenced by the practically achievable statistical uncertainty, low-energy transport cut-off, and the number of particle histories. The FSBP algorithm uses the EPL [7, 8] for density correction similar to RT, and applies mass density lateral scaling corrections and kernel density correction factors [9]. Of the three algorithms, RT coupled with fixed cone is commonly used for CyberKnife MBM SRS. Use of the MC algorithm is gaining traction due to improved computing efficiency.

Commissioning of TPSs should be thorough and exclude systematic errors as much as practically possible [10]. Furthermore, the TPS commissioning tests should simulate a wide range of techniques and scenarios that represent the intended clinical applications [11–16]. Due to several potential errors with MLC based delivery, the commissioning process is more rigorous compared to fixed cone beam collimation. Commissioning tests for fixed cone beam collimation SRS include verifying: Tissue Maximum Ratio (TMR) or Tissue Phantom Ratio (TPR), Output Factors (OF), machine output calibration, end-to-end testing, imaging, etc. These tests are limited for MBM SRS. A retrospective study by Mukwada et al. [17] demonstrated that MBM SRS is influenced by a combination of factors, including proximity of gross tumour volumes (GTVs), the number of GTVs, the size and geometric distribution of GTVs, beam orientations, and the use of multiple prescription dose levels (MPDLs). These aspects should be thoroughly investigated at commissioning.

The focus of the present work is on tests that can uncover inaccuracies in MBS SRS due to the treatment planning algorithms. Conventional commissioning tests for cone-based SRS are well established and are outside the scope of this study.

## Methods

**Fig. 1:**
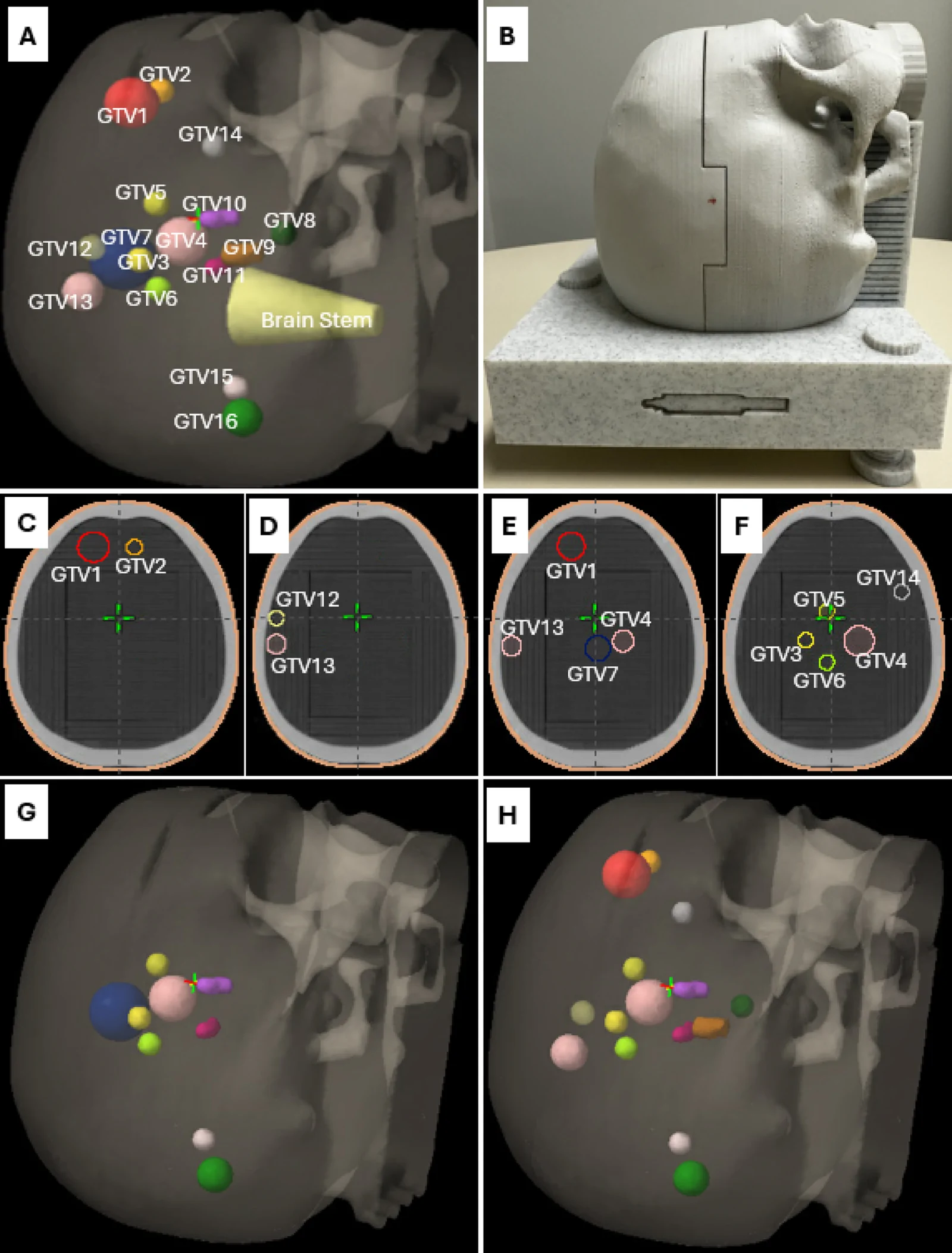
Distribution of GTV1 to GTV16: **A** Phantom showing geometric distribution of GTVs, **B** Phantom image [18], **C** Plan 1 GTVs, **D** Plan 2 GTVs, **E** Plan 3 GTVs, **F** Plan 4 GTVs, **G** Plan 5 GTVs and **H** Plan 6 GTVs. **C** and **D** show the GTVs on axial slices. Both **G** and **H** show GTVs in 3D view and the GTV numbers can be deduced from **A**

An in-house designed phantom [18] was computed tomography (CT) scanned following the departmental SRS scanning protocol (1 mm slice thickness, 120 kV, large field of view, and the FC9 reconstruction algorithm) using a Toshiba Aquilion CT scanner (Canon Inc., Tokyo, Japan). The scan was imported into the Eclipse TPS (Varian Medical Systems, Palo Alto, USA), and sixteen non-overlapping GTVs were contoured (Fig. 1). Further explanation is provided in the test cases below.

The contoured dataset was imported into the Precision TPS (Accuray Inc., Sunnyvale, USA) for CyberKnife treatment planning. Six treatment plans were generated, as shown in Tables 1 and 2, with the dose calculated using the RT algorithm on high resolution grid. On the same phantom, QA plans were generated and delivered using the M6 CyberKnife system. Keeping the monitor units (MUs), nodes, and number of beams fixed, each plan was recalculated using the MC algorithm on high resolution dose calculation grid and 1% uncertainty. RT and MC calculated coronal or sagittal dose planes, corresponding to film positions, were exported from the TPS for film dosimetry assessments. The gamma criteria of 95% pass at 3%/1 mm, with 10% threshold was used as per our department protocol. However, the evaluation was also performed at a less stringent criteria of 3%/2 mm, 10% threshold.

**Table 1:** Planning details for the six test scenarios. Dose was prescribed to the isodose line (70–80%) covering the GTVs in that plan

Target	Description of target placement	Prescribed dose (Gy)	Target vol (cm^3^)	Plan and test number					
				Test 1	Test 2	Test 3	Test 4	Test 5	Test 5
GTV1	Forehead	8	4.1	Plan 1		Plan 3			Plan 6
GTV2	Forehead	20	0.51	Plan 1					Plan 6
GTV3	Mid	8	0.51				Plan 4	Plan 5	Plan 6
GTV4	Mid	8	4.14			Plan 3	plan 4	Plan 5	Plan 6
GTV5	Mid	20	0.5				Plan 4	Plan 5	Plan 6
GTV6	Mid	20	0.5				Plan 4	Plan 5	Plan 6
GTV7	Mid	15	8.14			Plan3		Plan 5	
GTV8	Mid/vertex	20	0.51						Plan 6
GTV9	Mid	8	0.71						Plan 6
GTV10	Mid	20	0.49					Plan 5	Plan 6
GTV11	Mid	8	0.44					Plan 5	Plan 6
GTV12	Rt	20	0.51		Plan 2				Plan 6
GTV13	Rt	10	1.72		Plan 2	Plan 3			Plan 6
GTV14	Lt	20	0.51				Plan 4		Plan 6
GTV15	Post	15	0.51					Plan 5	Plan 6
GTV16	Post	10	1.75					Plan 5	Plan 6

**Table 2:** Plan parameters for the 6 plans

Plans	Cone size (mm)	Number of beams	Number of nodes	Number of MUs	Treatment time (mins)
Plan 1	7.5, 10, 15	125	45	9360	44
Plan 2	5, 10	189	87	16,537	58
Plan 3	12.5, 20	224	107	12,018	61
Plan 4	7.5, 12.5	236	109	26,458	81
Plan 5	7.5, 12.5, 15, 20	261	129	38,327	109
Plan 6	7.5, 15	335	160	64,205	143

*Test 1:* Simple geometry, targets close to each other and away from the skull, with MPDLs.

Plan 1 had two targets of different sizes located close to each other and away from the skull (Fig. 1C), with different prescribed doses. The test assessed the accuracy of dose calculation for a simple geometry, two levels of prescribed dose, in a homogeneous medium.

*Test 2:* Simple geometry, targets close to each other and close to the skull, with MPDLs.

Plan 2 had two different sized targets close to the skull (Fig. 1D), with different prescribed doses. The test assessed the dose accuracy in areas of inhomogeneity and the impact of MPDLs for targets close to each other.

*Test 3:* Large-spaced targets, large closely spaced targets with MPDLs.

Plan 3 had four large targets. Two were widely spaced and two close to each other in the middle of the phantom (Fig. 1E). The plan had three levels of dose prescription. The test assessed the impact of MPDLs for large targets that are close to each other, while others are further apart.

*Test 4:* Targets close to each other, complex geometry, and MPDLs.

Plan 4 had four targets close to each other in the middle of the brain and one peripheral GTV (Fig. 1F). The targets were of three different sizes and the plan had two levels of dose prescription. The test assessed the impact of multiple targets close to each other on dose distribution.

*Test 5:* Several targets, some close to each other, complex geometry, and MPDLs.

Plan 5 had nine targets, with seven of them in the middle of the brain. The test assessed the algorithm performance in the presence of several targets, MPDLs, and complex geometry. There were two posterior targets simulating tumours posterior to the brain stem.

Plan 6 tested dose calculation accuracy for even more targets close to each other. To keep the total GTV volume within ± 2.5 cm^3^ of the recommended or trialled MBM SRS of 15 cm^3^ [19, 20], the largest GTV was excluded.

The six treatment plans were delivered to the phantom. For dosimetry, EBT4 films (Ashland Inc. Wayne, NJ USA) were inserted in respective GTV slots. The films were precisely cut using the Beambox laser cutter (Flux, Vic, Australia). The films were scanned after 16 h using an Epson 12000XL scanner (Seiko Epson Corporation, OR, USA) and analysed with an in-house developed film analysis software (Film Wizard) in absolute mode. All films were scanned under consistent scanner settings and orientation to minimise scanner-induced variability. The Film Wizard software corrects for “scan-to-scan” variations by performing colour correction against a reference image acquired during calibration. A film calibration curve was generated by delivering known dose to the film and then assigning the dose to the corresponding scanned 16-bit, three-channel pixel value. The Film Wizard software was already validated against ionisation chamber measurements and the commercially available SNC patient dose analysis software version 7.6. (Sun Nuclear Corporation, USA). In cases where the analysis appeared inconsistent, the dose planes were determined and exported by an independent physicist. This eliminated potential errors arising from the use of non-corresponding dose planes.

## Results

**Fig. 2:**
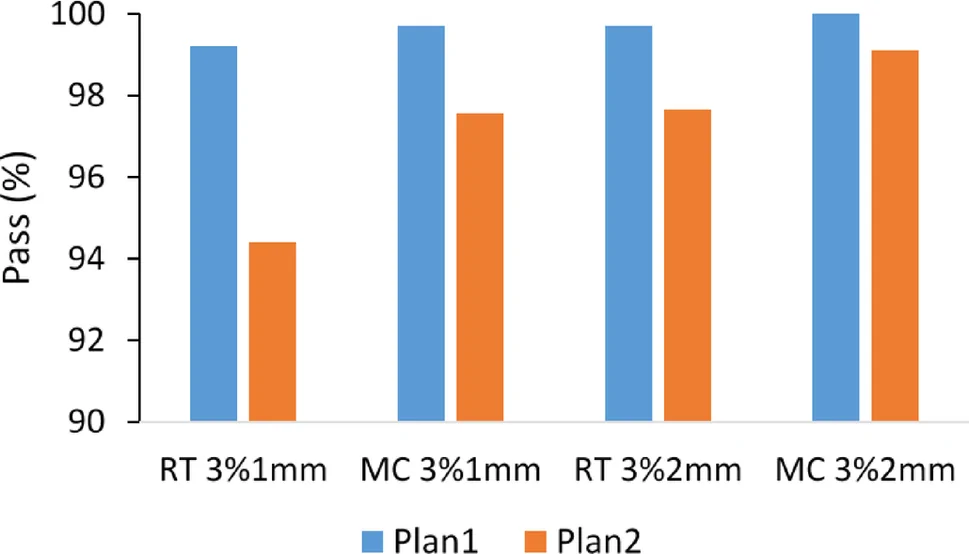
Absolute dose gamma pass rates for plan 1 and plan 2. Due to a single dose plane analysis per plan the bar chart was used

**Fig. 3:**
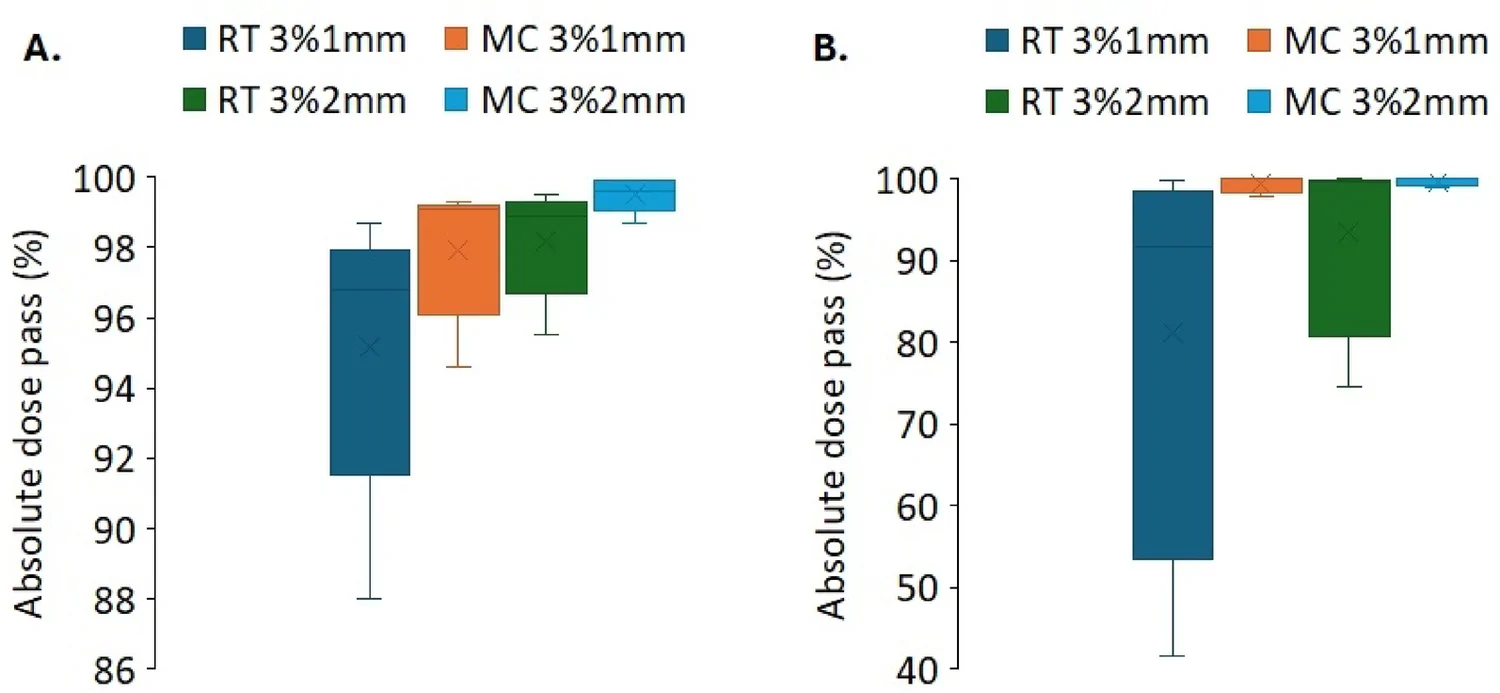
Box plots for **A** plan 3 and **B** plan 4. The box plots were created from the analysis of five dose planes for plan 3 and four for plan 4. All the GTVs and the out-of-field (OOF) areas were analysed as they were covered by one of the dose planes at a minimum

**Fig. 4:**
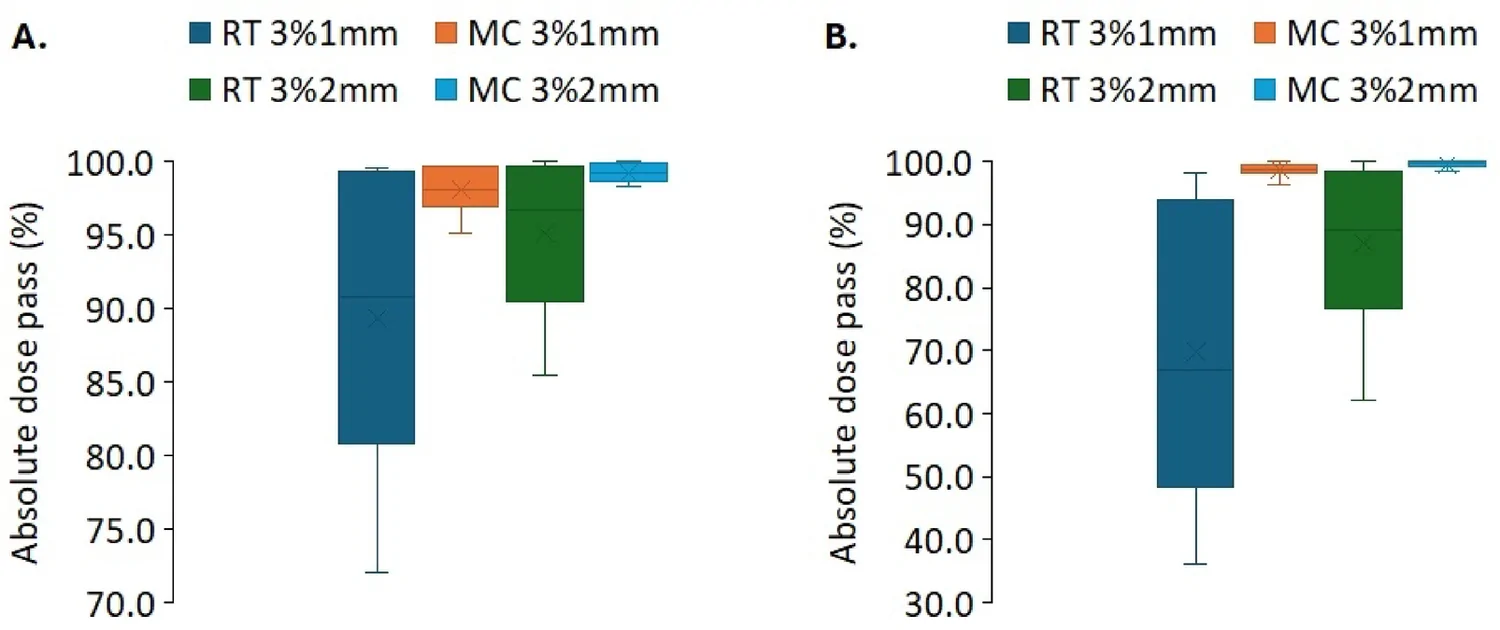
Box plots for **A** plan 5 and **B** plan 6. The box plots were created from the analysis of six dose planes for plan 5 and eight for plan 6. All the GTVs and the OOF areas were analysed as they were covered by one of the dose planes at a minimum

**Fig. 5:**
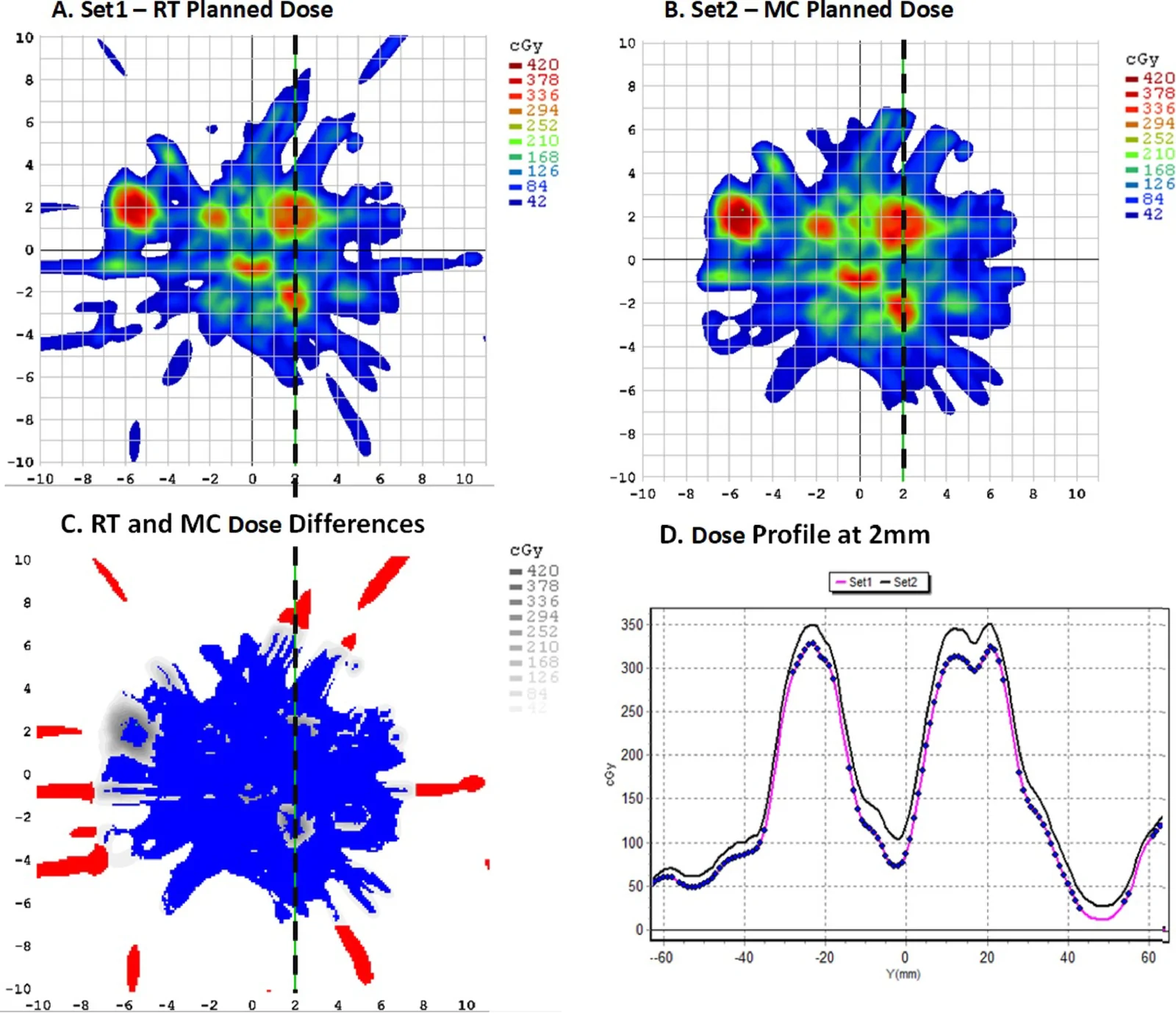
Planned RT and MC dose comparison for plan 6 worst case dose analysis. In **A**, **B** and **C**, the X-axis is horizontal and Y-axis is vertical and both are in cm. In the dose overlay (**C**), blue indicate areas where set 1 (RT) is colder than set 2 (MC), and red is where set 1 is hotter than set 2. The dose profile in **D** was taken parallel to the Y-axis

Results of film dosimetry analyses are given in Figs. 2, 3, 4 and 5.

Results for test cases 1 and 2 are shown in Fig. 2.

In test 1, plans created with both RT and MC dose calculation algorithms showed over 99% gamma pass rate for the 3%/1 mm criteria, while in test 2, the gamma pass rates for 3%/1 mm criteria were 94.4% and 97.7% for RT and MC, respectively. The 3%/2 mm criteria gave 97.6% and 99.1% gamma pass rates for the RT and MC algorithms, respectively.

Results of tests 3 and 4 are illustrated in Fig. 3. All GTVs had over 95% pass rate for the 3%/1 mm gamma criteria except GTV1 which showed 88% (RT) and 94.6% (MC) pass rates (Fig. 3A). GTV1 had more beams passing near or through it delivering higher dose to GTV7.

For plan 4 (test 4), the MC plans achieved over 97.8% pass rate for the 3%/1 mm criteria (Fig. 4). The RT plans did not pass either of the 3%/1 mm and 3%/2 mm gamma criteria and the difference from MC was more than 50% for the worst case as shown in Fig. 3B. Close targets had lower gamma pass rates. RT dose planes that failed had MPDLs and GTVs in close proximity.

In Test 5, MC passed both gamma criteria for plan 5 and 6 with minimum pass rate of 95.1% as shown in Fig. 4A, B. Compared to MC, the worst-case RT pass rate for plan 5 was 72% (3%/1 mm) and 36.2% (3%/1 mm) for plan 6. Further investigation of plan 6 confirmed the observed differences between MC and RT planned doses, (no measurement, Fig. 5). The MC algorithm calculated more dose than RT algorithm in the GTVs and the peripheral, and in some cases, the dose difference was more than 30 cGy, as shown in Fig. 5. The RT plan failed mostly in the middle of the brain, where several GTVs were in proximity and MPDLs were applied.

## Discussion

This study demonstrated tests that can expose the limitations of dose calculation algorithms in MBM SRS. The tests varied from a few to 15 MBMs, MPDLs, varying distances between GTVs, and increasing complexity in the spatial distribution of GTVs. The precision RT and MC algorithms were assessed using film dosimetry and gamma criteria of 95% pass at 3%/1 mm and 3%/2 mm. Gamma tolerances used align with the tighter tolerances recommended for SRS [5, 10]. The assessment included the GTVs and significant areas around the GTVs thereby evaluating both in-field and OOF dose. For simple and fewer MBM, the performance of both algorithms was acceptable. As the number of GTVs increased and the plans became more complex, the RT plans failed while the MC plans consistently scored high against the gamma pass criteria. Although some gamma criteria have been criticized for being insensitive and failing to detect significant clinical errors [10, 21–26], gamma remains the most widely used method to compare planned and measured doses [25–27]. In addition, SRS mandates strict target delineation guidelines and stringent tolerance to dose objectives to OAR such as the brain stem. The choice of MBM SRS instead of whole brain radiation therapy is to reduce toxicity and maintain better neurocognitive function. Inaccuracies in dose prediction and assessment may result in treatment failure, undesired toxicity and higher doses to OAR [28]. Adherence to robust dose calculation algorithms and appropriate patient specific quality assurance approaches should aid in curbing over or under radiation dosage.

In plan 1 (Test 1), which was a simpler MBM SRS case, the RT and MC algorithms performed equally well. The GTVs were in a homogeneous area, and the two levels of prescription seemed to have less impact. The agreement between RT and MC plans and measured films also suggests that the beam models were performing well, especially the RT algorithm which interpolates and extrapolates the measured data and does not fully account for scatter.

Test 2 evaluated dose accuracy in areas of inhomogeneity and the impact of MPDLs for targets close to each other. Plan 2 had targets close to the skull, and the RT algorithm pass rate notably decreased for tighter gamma criteria compared to MC dose calculation. Empirical dose calculation algorithms are unable to account for the impact of the bone inhomogeneity near the GTVs. Therefore, targets close to the skull require careful dose assessment, as the algorithm performance may be suboptimal.

Test 3 evaluated the algorithms for larger GTVs at large and small distances from each other, using MPDLs. Fewer GTVs close to each other with MPDLs did not show large differences in gamma pass rates. However, GTV1 which had the lowest prescribed dose (8 Gy) and was further away from other GTVs, showed the lowest pass rate. This was attributed to possible increased unaccounted scatter dose from the several beams passing through or near GTV1, contributing to the dose in GTV7 (15 Gy). In addition, there is uncertainty in the OOF dose due to beam modelling limitations and measured OCR data inaccuracies that may be contributing to the dose error. Several studies demonstrated that TPSs tend to underestimate dose in the OOF region [29–34]. Although the measured data used in this study was validated by using the microdiamond and the vendor consensus data, there is uncertainty in the measured data due to changes in the beam spectrum and dose rate in the OOF region. For example, the mean energy (in-field) for a 6MV photon at the depth of max dose is 1.6 MeV and the OOF mean energy is 0.2–0.6MeV [30]. Understanding the algorithm limitations, beam orientations, and prescription dose levels could help reduce undesired dose delivery.

Test 4 evaluated five GTVs, with four central GTVs proximal to each other, and one in a peripheral location. The consistent agreement between MC plan and measurements when the GTVs were close with MPDLs applied (Fig. 3B) demonstrates the ability of MC algorithm in tracking individual particle interaction until the energy is deposited. On the other hand, the RT algorithm performed poorly when more GTVs were close to each other and MPDLs applied. In this complex plan, failure to fully account for scatter dose and uncertainty in OOF dose became more pronounced.

Test 5 evaluated the compounded effect of several GTVs, their proximity, complexity of geometry, and MPDLs. Some of the GTVs were close to each other, while a few were further apart, and there were four levels of dose prescription (8 Gy, 10 Gy, 15 Gy, and 20 Gy). MPDLs are usually used to address dose to OARs, the impact of large GTV volumes, and the presence of several GTVs [11, 35–37]. In our department, a two-level dose prescription is commonly used, and the difference between the prescriptions ranges from 2 to 5 Gy. Rarely is three prescription dose levels used, and four levels tested the extreme case. In test cases 5 (9 GTVs) and 6 (15 GTVs), the MC plans performed superior to RT plans. The presence of multiple GTVs in complex geometry with MPDLs could lead to more scatter dose, especially to GTVs with lower levels of prescribed dose. To achieve accurate plans for such cases, measured OCR and beam modelling uncertainty should be reduced and the scatter dose should be fully accounted for.

Across all plans, the RT algorithm predicted less dose compared to MC and measured. The comparison of RT and MC calculated dose planes (excluding measurements) for the failing cases confirmed the observed differences with measurements. Figure 5 shows the disagreement between RT and MC algorithms both in-field and OOF. The MC plan agreed with measurement confirming the robustness of the algorithm [5]. The authors hypothesise that, the RT algorithm calculates dose without fully accounting for scatter and the uncertainty in the OOF dose; hence the MUs will be higher, delivering more dose than predicted. The situation is exacerbated when more GTVs are involved, and when they are close to each other.

It can be deduced from Fig. 5 that QA analysis should be performed in absolute dose. If relative dose were used in Fig. 5 and in the discussed QAs, then the pass rate would have drastically increased and would appear as though the plan had passed QA. Yet, the analysis in relative mode would have masked the dose mismatch between planned and delivered. In addition, it was observed that the 3%/2 mm is a loose tolerance that allows less optimal treatment plans and delivery in SRS. Overall, SRS is characterised by precise high dose delivery and stringent planning tolerance, which should be matched by stringent QA tolerance. Furthermore, the QA results of the same plan demonstrate variation in algorithm performance across GTVs within a single patient. It follows that those who perform QA measurements on representative GTV(s) for plan check should pick those that are prone to having errors such as GTVs with lower prescription and have more beams passing through them.

### Limitations

The study assumes that the RT and MC beam models were optimal. The data used for beam modelling was compared with the vendor’s golden data and against measured data with different detectors and agreed within tolerance. Hence, the deficiencies were more inclined to lie with the performance of the algorithm.

## Conclusion

Commissioning of treatment planning algorithms for simple and few brain metastases SRS should not be assumed to hold for MBM SRS. The RT algorithm was found unsuitable for complex MBM SRS. Increase in the number of GTVs and their proximity is associated with an increase in OOF dose uncertainty and scatter dose which if unaccounted for will result in dose errors. Complex GTV geometrical distribution and MPDLs may require more beams from the same direction contributing dose to other GTVs especially to those with lower dose prescription. For very complex scenarios, significant dose differences both in-field and OOF can be observed hence the GTV and health brain dose should be assessed where possible. Representative GTV(s) for plan QA should be carefully chosen as some delivery errors might be missed if a QA GTV is poorly selected. The impact of multiple GTVs, their proximity, geometrical distribution, and MPDLs on the calculated and delivered dose should be thoroughly evaluated.

## Data Availability

The data analysed during the study is available from the corresponding author upon reasonable request.
